# Validity of self-measured waist and hip circumferences: results from a community study in Malaysia

**DOI:** 10.1186/1475-2891-12-135

**Published:** 2013-10-05

**Authors:** Daniel D Reidpath, Julius Chee-Ho Cheah, Fui-Ching Lam, Shahjahan Yasin, Ireneous Soyiri, Pascale Allotey

**Affiliations:** 1 South East Asia Community Observatory (SEACO), School of Medicine and Health Sciences, Monash University, Bandar Sunway, Selangor DE, Malaysia

## Abstract

**Background:**

Measures of central adiposity are better predictors of adverse health events than BMI. Nonetheless, BMI is more widely used in health research. One reason for this may be the limited research supporting the self-measurement of waist and hip circumference. The lack of validity studies is particularly acute in Asia. The main objective was to establish the validity of self-measurement of waist and hip circumference in a community setting and the correlation of those measures with BMI, blood pressure, and blood glucose levels.

**Methods:**

A community based, cross-sectional survey. A “healthy living expo” at a shopping mall in a rural town on peninsular Malaysia One hundred and thirty six (136) individuals volunteered to participate in the study, 125 of whom met the inclusion criteria. The ethnic distribution of the participants was 80% Chinese, 17% Malay and 3% Indian. Most participants were female (60%), with participants’ ages ranging from 18 to 78 years (mean, 47.2). Self and assisted measurements of waist and hip were taken. Blood pressure, non-fasting blood glucose, height, and weight were also measured. Bland Altman plots and Lin’s concordance coefficient were used to measure agreement between self and assisted measures. Pearson’s correlation was used to examine the association of self and assisted measures with blood pressure, blood glucose, and BMI.

**Results:**

There was a downwards bias in self measured waist (-0.81 cm) and hip (-1 cm) circumferences compared with assisted measures. The concordance for the self and assisted measures of waist, hip and the ratio of the two were, respectively, .96, .93 , and .84. The correlation between measures of central adiposity and BMI, blood pressure and blood glucose were similar for self and assisted measures.

**Conclusion:**

The results provide additional support for the use of self-measurement of waist and hip circumference studies of central adiposity, but is limited by the specificity of the setting.

## Introduction

Overweight and obesity (excess adiposity) are associated with an increased risk of diabetes, arthritis, cardiovascular disease, and certain cancers [1-3]. Over the past twenty years the prevalence of overweight and obesity has risen sharply in many countries [4-7]; and cheap, straightforward techniques for population surveillance of adiposity remain critical. In large epidemiological studies measuring adiposity almost always relies on proxy measures such as the body mass index (BMI) [8].

There is evidence, that proxy measures of central adiposity – waist circumference (WC) and waist to hip ratio (WHR) – are better predictors of adverse health events, including mortality, than BMI [9-11]. A tape measure is also an easier piece of equipment to carry into the field than a stadiometer and a set of weighing scales. Notwithstanding these advantages BMI remains the adiposity metric of choice in most medical research. In 2011, for instance there were 13,909 papers listed in PubMed related to BMI and less than one fifth as many papers related to WC or WHR (n = 2,422).

One factor affecting the adoption (or lack of adoption) of WC or WHR may be the current reliance on inexpensive self-reported BMI measures, and the concomitant uncertainty among the research community about the validity of self measured waist and hip data. Self-reported height and weight, which is used to estimate BMI, has been widely used e.g., [12,13]; and the validity and biases associated with self-reported height and weight have been the subject of considerable research since at least the 1980s [14]. These have included, for instance, validity studies from Asia [15-17], North America [18], Central America [19], and Europe [20].

In contrast there are relatively few validity studies of self measurement associated with WC and WHR, and with one exception [15], the studies all appear to have come from Europe and North America [21-31]. The lack of validation studies on self measurement of WC and WHR from different populations with different anthropometry, cultural practices, and levels of education will necessarily affect the adoption of WC and WHR as alternative measures of adiposity in medical research. The generalisability of the studies are further affected by variations in the choice of tape measure including paper [22,30,31], cloth or plastic [21], marked or unmarked [25], or constant tension tape measures [28]; differences in the device used by participants and technicians [21]; the instructions provided; the presence or absence of light clothing; and the sampling frame (clinical, community, occupational, random, convenience, and so forth).

In this research, we contrasted self measurement and assisted measurement of waist and hip circumference and WHR in a community-based sample from a district town in peninsular Malaysia, using a now standard protocol [32]. We also examined the validity of the measures for scientific research; specifically we examined the extent to which the measurement approach affected statistical relationships with BMI, diastolic and systolic blood pressure, and non-fasting blood glucose. While the need for validation is critical, the research was relevant also to explore the feasibility of self measurement within a context where the permissibility of data collectors taking these measurements is constrained by cultural and social mores.

## Methods

### Ethics

Participation in the validation study was voluntary and informed, written consent was obtained from participants. The research was approved by the Monash University Human Research Ethics Committee.

### Participants

Participants were a convenience sample of 136 people attending a “healthy living expo” at a shopping mall in the rural town of Segamat in Johor state, Malaysia. Of the 136 participants, usable waist and hip measurements were obtained from 125 participants. The ethnic distribution of the participants was 80% Chinese, 17% Malay and 3% Indian. Most participants were female (60%), with participants’ ages ranging from 18 to 78 years (mean, 47.2). The sample size fell within the recommended, cost effective, range of 100 to 200 participants for agreement studies [20].

Participants were part of a community health screening exercise offered at the shopping mall by staff and medical students from the South East Asia Community Observatory (SEACO), Monash University Sunway Campus, and staff from the district office of the Ministry of Health. Health screening was open to all members of the public.

### Devices

Waist and hip circumferences were measured using constant tension measuring tapes (model: Orbitape). Constant tension measuring tapes reduce the individual variation in how tight the tape is pulled to determine circumference. Non-fasting, capillary blood glucose measurements were made using electronic glucometers (model: Omron Healthcare HEA-220).

### Procedure

Whether or not a person chose to participate in the research, the procedure for all people attending the health screening was identical, except that data from non-participants did not contribute to the final data set. Screening began with the registration process to record demographic information that included age, sex, and ethnicity. After registration, participants were shown a video and a live demonstration on the proper method to conduct self-measurement of waist and hip circumference with the Orbitape. The World Health Organization STEPwise protocol for measurement was used: WC was measured around the midpoint between the lower margin of the last palpable rib and the top of the iliac crest; hip measurement was taken at the maximum circumference over the buttocks [32]. Participants then proceeded to a private curtained area where they self-measured their own hip and waist. After recording their results, the same measurements were taken (blinded) by medical students. All measurements were taken once over light clothing and values were recorded in centimetres. Participants proceeded to other screening stations where height and weight, blood pressure, and non-fasting capillary blood glucose measurements were made by health staff and trained medical students.

### Data analysis

The level of agreement, or concordance, between the self and assisted measures were examined using graphical techniques including Bland Altman plots, [33] and formally tested using Lin’s concordance correlation coefficient [34]. The degree to which the assisted and self measured waist, hip and WHR correlated with other anthropometric or health outcome measures was examined using the Pearson’s product moment correlation coefficient [34].

## Results

Summary results for the self and assisted measurement of waist, hip, and waist to hip ratio are shown in Table 1.

**Table 1:** Summary statistics for self (s) and assisted (a) measurement of waist, hip, and waist to hip ratio

	**Min**	**Max**	**Median**	**Mean**	**SD**	**n**
WC (s)	64.4	117.9	86.6	87.7	10.79	125
WC (a)	64.5	116.5	87.6	88.2	10.93	125
Hip (s)	83.5	136.8	97.5	98.4	8.72	125
Hip (a)	80.5	137.0	98.5	99.5	8.5	125
WHR (s)	.75	1.11	.88	.89	.072	125
WHR (a)	.72	1.13	.88	.89	.073	125

The minimum, median, and mean WC values for self and assisted measurements were within 1 cm of each other – the maximum was within 1.5 cms. The summary statistics for hip measurement were similarly close. Summary statistics for waist to hip ratios also showed little divergence.

**Figure 1:**
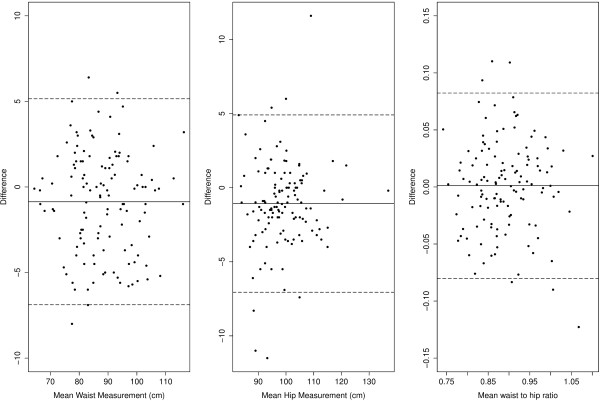
Bland Altman plots for waist, hip, and waist to hip ratio.

Bland–Altman plots illustrate the difference between two measures against the average value of the two measures [33]. 'Agreement’ is related to both the mean difference between the self and assisted readings, and the amount of variation in the differences. Figure 1 shows the Bland Altman plots for the self and assisted measures of waist and hip circumference and the derived measure of waist to hip ratio. The solid horizontal line indicates the mean difference between the measures. The dashed horizontal lines show the 95% limits of agreement around the mean difference.

Most of the points lie comfortably within the 95% limits of agreement. For the plot of hip measurement, however, there appear to be some extreme outliers showing differences between the assisted measurement and the self-measurement in excess of 10 cms. The data were rechecked for obvious recording errors, but none were apparent. The differences could not be explained by a systematic difference between self- and assisted-measures. In two cases the assisted measure was more than 10 cm greater than the self measurement, and in the other case the self measurement was more than 10 cm greater than the assisted measurement.

The mean differences between the self and assisted measures of waist (-0.81 cm) and hip (-1 cm) circumference indicated a bias, with self-measurement on average lower than assisted measurement. In both cases the mean of the differences was significantly different from 0 (waist: t = -3.22, df = 124, p = .002; hip: t = -4.00, df = 124, p = 0.0001). The 95% limits of agreement – the variation in the differences – for the waist (-0.87 ±6.02 cms ) and hip (-1.1 ±5.98 cms ) were around ±6 cms, with the 68% limits of agreement around ±3 cms. The mean of the differences in the WHR showed a slight, non-significant bias (mean difference = .001, p = .76). Most of the points lie comfortably within the 95% limits of agreement, although there was one point that lay well below the lower limit and a couple of points lay well above the upper limit. The concordance correlation coefficient for the waist, hip and WHR were, respectively, 0.96 (95% CI: .94 –.97); .93 (95% CI: .91–.95); and .84 (95% CI: .78–0.89).

Ideally, self-measurement and assisted measurement would agree perfectly. Given that they do not agree perfectly, the question of sufficiency necessarily arises. Is self-measurement a sufficiently good measure to replace assisted measurement? One approach to considering this question is to evaluate the relationship between each measure and some health outcome of interest; i.e., in research would the relationships between the measures and potential outcomes of interest be similar? Table 2 shows the Pearson’s product moment correlation between the self and assisted measurement of waist, hip, and waist to hip ratio, and BMI, blood pressure, and blood glucose.

**Table 2:** Pearson correlation coefficients between self (s) and assisted (a) measures of waist, hip and waist to hip ratio, and BMI, systolic and diastolic blood pressure, and non-fasting capillary blood glucose

	**BMI**	**BP**	**BP**	**Non-fasting**
		**Systolic**	**Diastolic**	**Blood glucose**
WC (s)	.83 (.76–.88)	.29 (.12–.44)	.30 (.13–.45)	.35 (.17–.50)
WC (a)	.87 (.82–.91)	.30 (.13–.45)	.32 (.15–.47)	.35 (.17–.50)
Hip (s)	.88 (.83–.91)	.22 (.04–.38)	.26 (.09–.42)	.26 (.08–.43)
Hip (a)	.88 (.83–.91)	.20 (.03–.37)	.33 (.16–.48)	.25 (.06–.42)
WHR (s)	.34 (.17–.49)	.21 (.04 –.37)	.18 (.00– .34)	.24 (.05–.41)
WHR (a)	.43 (.27–.56)	.24 (.07 –.40)	.14 (-.04 –.31)	.27 (.08–.44)

Parenthetically, 95% confidence intervals are also shown.

With the exception of the correlation between the assisted measurement WHR and diastolic blood pressure, all correlations were statistically significant (p < .05). Generally the assisted and self-measured correlation coefficients provided very similar estimates of the relationships’ with BMI, blood pressure, and blood glucose; and never significantly different from each other.

## Discussion

We sought to determine the extent to which a Malaysian community sample could provide accurate self measurements of waist and hip circumference and WHR, and the degree to which the self measurements could be used in studies of relationships with other health markers. The results of this study supported the use of waist and hip self measurement.

The measures had on average a small downward bias (around 1 cm), with 95% limits of agreement around ±6 cms around the downward biased estimate. The concordance for waist and hip measures was strong – 0.96 and 0.93 respectively. These results were similar or better than those reported by Lim and colleagues in their study of Thai students [15], and was broadly in keeping with other self measurement studies [22,30,31]. Also in keeping with those studies, WHR measurement was less accurate than WC or hip circumference measurement [29].

The greatest limitation of the study related to sampling. Like other community, occupational or university based studies where sampling was non-random, one is left to speculate about the generalisability of the findings [15,21,24,31]. Specifically, what is the underlying population represented by the sample? Notwithstanding this limitation, the study does contribute additional data to an otherwise extremely limited evidence-base of non-European or North American studies [21-31]. Like self-reported BMI, with its known biases, self measurement of waist and hip circumference should not be mistaken for a gold-standard, and should not form the basis for a clinical assessment. However, for population-based studies where there are issues of either cost or personal privacy, self measurement is a credible, if potentially weaker alternative to assisted measurement.

## Conclusions

The results suggest that self measurement of waist and hip circumference using constant tension measuring tapes provide a favourable alternative for population surveillance of central adiposity in a community setting. The relationships between self and assisted measures with other health markers were generally very similar.

## Competing interests

The authors declare that they have no competing interests.

## Authors’ contributions

DDR Conceived the study, supported the design and analysis, and wrote the first draft; JCHC Supported the design of the study, and the data collection, and contributed to the writing. FCL Supported the design of the study and managed the analysis; SY Conceived the study, supported the data collection, and edited; IS supported data collection and analysis and edited; PA conceived the study, supported the design, and data collection, and edited. All authors read and approved the final manuscript.
